# Significance of hypergammaglobulinemia in patients with chronic myelomonocytic leukemia

**DOI:** 10.1007/s10354-022-00983-6

**Published:** 2022-11-29

**Authors:** Marie-Therese Zack, Klaus Geissler

**Affiliations:** 1 Medical School, Sigmund Freud University, Vienna, Austria; 2 Department of Internal Medicine V with Hematology, Oncology and Palliative Care, Hospital Hietzing, Wolkersbergenstr. 1, 1130 Vienna, Austria

**Keywords:** Chronic myelomonocytic leukemia, Hypergammaglobulinemia, Survival, Chronic inflammation, NRAS, Chronische myelomonozytäre Leukämie, Hypergammaglobulinämie, Überleben, Chronische Entzündung, NRAS

## Abstract

Chronic inflammation is often indicated by a relative increase in the gamma globulin fraction in the serum electrophoresis. In a retrospective study, we analyzed the prevalence of relative hypergammaglobulinemia in 60 patients with chronic myelomonocytic leukemia (CMML), its potential prognostic impact, and potential correlations with laboratory and molecular features. Relative hypergammaglobulinemia (> 20%) was found in 25/60 (42%) patients. The median survival of patients with relative hypergammaglobulinemia was significantly shorter than in patients without hypergammaglobulinemia (10 vs. 24 months, *p* = 0.018). There was no difference between the groups regarding leukocyte count, hemoglobin value, and platelet count, but a higher prevalence of *NRAS* mutations and a lower prevalence of *ZRSR2* mutations in patients with hypergammaglobulinemia. Our results show that hypergammaglobulinemia is present in a proportion of CMML patients and that this abnormality is associated with poor overall survival. The role of chronic inflammation in the pathophysiology of CMML needs to be further investigated.

## Abstract (de)

Eine chronische Entzündung wird häufig durch eine relative Vermehrung der Gammaglobulinfraktion in der Serumelektrophorese angezeigt. In einer retrospektiven Studie wurde die Prävalenz einer relativen Hypergammaglobulinämie bei 60 Patienten mit chronischer myelomonozytärer Leukämie (CMML) analysiert, ihre potenzielle prognostische Bedeutung sowie eine mögliche Korrelation mit laborchemischen und molekularen Parametern. Eine relative Hypergammaglobulinämie (> 20 %) lag bei 25/60 (42 %) CMML-Patienten vor. Das mediane Überleben von Patienten mit relativer Hypergammaglobulinämie war signifikant kürzer als jenes von Patienten ohne Hypergammaglobulinämie (10 vs. 24 Monate; *p* = 0,018). Keine Unterschiede zwischen beiden Gruppen wurden für Leukozytenzahl, Hämoglobinwerte und Thrombozytenzahl festgestellt jedoch eine höhere Prävalenz von *NRAS*-Mutationen und eine geringere Prävalenz von *ZRSR2*-Mutationen bei Patienten mit Hypergammaglobulinämie. Die vorliegenden Ergebnisse zeigen, dass eine relative Hypergammaglobulinämie bei einem Teil der CMML-Patienten vorliegt und dieser auffällige Befund mit einem schlechteren Überleben verbunden ist. Die Rolle einer chronischen Entzündung in der Pathophysiologie der CMML muss noch weiter untersucht werden.

## Introduction

Chronic myelomonocytic leukemia (CMML) is a rare, genotypically and phenotypically heterogenous hematologic malignancy of elderly people with an intrinsic risk to progress and transform into secondary acute myeloid leukemia (AML). With regard to the presence of myeloproliferation, CMML was originally subdivided into myeloproliferative disorder (MP-CMML; white blood cell count [WBC] count > 13 × 10^9^/L) versus myelodysplastic syndrome (MD-CMML; WBC count ≤ 13 × 10^9^/L MD-CMML) by the FAB criteria [1, 2]. Since CMML is characterized by features of both MDS and MPN, the World Health Organization (WHO) classification of 2002 assigned CMML to the mixed category, MDS/MPN [3]. CMML is further subclassified by WHO into three groups based on blast equivalents (blasts plus promonocytes) in peripheral blood (PB) and bone marrow (BM) as follows: CMML‑0 if PB < 2% and BM < 5% blast equivalents; CMML‑1 if PB 2–4% or BM 5–9% blast equivalents; and CMML‑2 if PB 5–19% or BM 10–19% blast equivalent, and/or Auer rods are present [4]. CMML patients may have a highly variable outcome, suggesting that several factors can determine the course of disease and the cause of death in these patients [5–9]. There are a number of established prognostic parameters that have been incorporated into several prognostic models [10–21]. In clinical practice, chronic inflammation is often indicated by a relative increase in the gamma globulin fraction of the electrophoresis. The potential contribution of chronic inflammation to the clinical outcome of CMML patients is poorly investigated. Using the database of the Austrian Biodatabase for Chronic Myelomonocytic Leukemia (ABCMML), we analyzed 60 CMML patients in whom serum electrophoresis was available in patient records.

## Patients and methods

### Patients

Recently, we have shown that the ABCMML may be used as a representative and useful real-life data source for biomedical research [22]. In this database, we retrospectively collected epidemiologic, hematologic, biochemical, clinical, immunophenotypic, cytogenetic, molecular, and biologic data of patients with CMML from different centers. The diagnosis of CMML and leukemic transformation was according to the WHO criteria [2, 3]. Clinical and laboratory routine parameters were obtained from patient records. A detailed central manual retrospective chart review was carried out to ensure data quality before analysis of data from institutions. Due to the fact that CMML may be considered as an evolutionary process from clonal hematopoiesis of indeterminate potential (CHIP) to CMML-related AML [23], and the fact that the distinction between mature and immature monocytic cells, which is required to determine the time of transformation into AML, is notoriously difficult due to the lack of reliable immunophenotypic markers, we found it more appropriate not to exclude the few CMML patients with transformation from our analysis [24].

In 60 CMML patients collected between 01.01.1990 and 31.03.2019, serum electrophoreses was available for analysis. This research was approved by the ethics committee of the City of Vienna on 10 June 2015 (ethics code: 15-059-VK).

### Molecular studies

Genomic DNA was isolated from mononuclear cell (MNC) fractions of the blood samples according to standard procedures. The mutational status of CMML-related protein-coding genes was determined by targeted amplicon sequencing using the MiSeq platform (Illumina, San Diego, CA, USA). Details of the gene panel, library preparation, and data processing have been reported previously [22]. Only variants with strong clinical significance according to the Standards and Guidelines for the Interpretation and Reporting of Sequence Variants in Cancer and VAF ≥ 5% were used for statistical analysis regarding a potential predictive value in various treatment options.

### Statistical analysis

The log-rank test was used to determine whether individual parameters were associated with overall survival (OS). OS was defined as the time from sampling to death (uncensored) or last follow-up (censored). Dichotomous variables were compared between different groups with the chi-square test. The Mann–Whitney U test was used to compare two unmatched groups when continuous variables were nonnormally distributed.

To measure the degree of relationship between protein fractions of serum electrophoresis, Pearson correlation was performed. All results were considered significant at *p* < 0.05. Statistical analyses were performed with SPSS v. 27 (IBM Corp., Armonk, NY, USA); the reported *p*-values were two-sided. The normal upper limits for gamma globulin, α1, and α2 fractions were 20%, 6%, and 12%, respectively. The normal lower limit for the albumin fraction was 50%.

## Results

### Characteristics of patients

The baseline characteristics of the 60 patients with CMML are shown in Table 1. In order to make comparisons with other published CMML cohorts possible, the percentages of patients regarding established prognostic parameters are given [17]. As seen in other CMML series, there was a male predominance in study patients and more than half of the patients were aged 70 years or older [17]. More than half (51%) of the study patients had leukocytosis > 13 G/L, which is more frequent as compared to other cohorts, where this proportion is usually below 50%. Two patients in this cohort have already transformed into CMML-related acute myeloid leukemia (AML).

**Table 1:** Characteristics of chronic myelomonocytic leukemia patients

	Cases *N* = 60	Percent (%)
*Age* *Evaluable* *=* *60*		
< 70 years	27	45
≥ 70 years	33	55
*Sex* *Evaluable* *=* *60*		
Male	35	58
Female	25	42
*Leukocytes* *Evaluable* *=* *59*		
> 13 G/L	32	54
≤ 13 G/L	27	46
*Hemoglobin* *Evaluable* *=* *59*		
< 10 g/dL	12	20
≥ 10 g/dL	47	80
*Platelets* *Evaluable* *=* *59*		
< 100 G/L	28	47
≥ 100 G/L	31	53
*Peripheral blood blasts* *Evaluable* *=* *55*		
Absent	42	76
Present	13	24

### Prevalence of hypergammaglobulinemia and other changes in serum electrophoresis in CMML patients

Relative hypergammaglobulinemia (> 20%) was found in 25/60 (42%) patients. A relative decrease of albumin < 50% was seen in 11/58 (19%) patients. The α1 and α2 fractions were increased in 18/60 (30%) and 9/60 (15%) CMML patients, respectively. There was a significant strong negative correlation between gammaglobulinemia and albuminemia (−0.821; *p* = 0.000), indicating the opposite behavior of these parameters. There was a significant weak negative correlation between gamma globulins and α2 fraction (−0.319; *p* = 0.017), whereas no significant correlation was observed between gamma globulins and the α1 fraction in the serum electrophoresis (−0.032; *p* = 0.815).

### Impact of relative hypergammaglobulinemia on survival

**Fig. 1:**
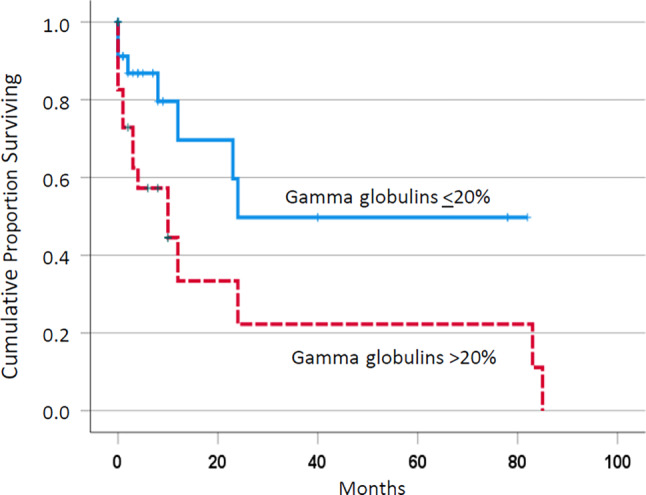
Kaplan–Meier plots for overall survival in chronic myelomonocytic leukemia patients with and without hypergammaglobulinemia

As shown in Fig. 1, the median survival of patients with relative hypergammaglobulinemia was significantly shorter than in patients without hypergammaglobulinemia (10 vs. 24 months, *p* = 0.018). Among established prognostic parameters including leukocytosis > 13 G/L, anemia < 10 g/dL, thrombocytopenia < 100 G/L, and the presence of blast cells in peripheral blood, only thrombocytopenia had an adverse impact on survival in the univariate analysis in the study cohort (Table 2). The significant effect of hypergammaglobulinemia in univariate analysis remained in the multivariate analysis in the presence of thrombocytopenia (Table 3).

**Table 2:** Univariate analysis of established single prognostic parameters in patients with chronic myelomonocytic leukemia

Factors	Factor present Median OS (months)	Factor absent Median OS (months)	*P*-value (Log-rank)
Gamma fraction > 20%	10.0	24.0	0.018
WBC > 13 × G/L	12.0	23.0	0.505
Hb < 10 g/dL	10.0	23.0	0.396
PLT < 100 × G/L	10.0	24.0	0.005
PB blasts present	12.0	12.0	0.633

The log-rank test was used to determine if individual parameters were associated with OS

*OS* overall survival, *WBC* white blood cell count, *Hb* hemoglobin, *PLT* platelet count, *PB* peripheral blood

**Table 3:** Hazard ratios, confidence intervals, and *p*-values of Cox regression analyses for survival including hypergammaglobulinemia and thrombocytopenia

Parameter	Hazard ratio	95% confidence interval	*P*-value
Gamma fraction > 20%	2.735	1.162–6.440	0.021
PLT < 100 G/L	3.365	1.376–8.229	0.008

*PLT* platelet count

### Laboratory and molecular features in the presence or absence of hypergammaglobulinemia

As shown in Table 4, there was no difference in the groups regarding laboratory parameters including leukocyte counts, hemoglobin values, platelet counts, and circulating blasts. Regarding molecular aberrations, mutations in the *NRAS* gene were more common in patients with hypergammaglobulinemia, whereas mutations in the *ZRSR2* gene were more common in patients without hypergammaglobulinemia (Table 5).

**Table 4:** Laboratory features stratified by the presence or absence of hypergammaglobulinemia

	All patients (*n* = 60)	Hypergamma+ (*n* = 25)	Hypergamma− (*n* = 35)	*P*-value
Age in years; median (range) Evaluable = 58	71 (36–91)	72 (36–84)	71 (44–91)	0.970
Sex (Male); *n* Evaluable = 58	35 (58%)	14 (56%)	21 (60%)	0.757
Leukocytes G/L; median (range) Evaluable = 57	14.9 (3–238)	20.8 (3–238)	14.1 (3.8–74)	0.212
Hemoglobin g/dL; median (range) Evaluable = 57	11.9 (7.6–15.0)	11.9 (7.5–14.8)	11.8 (7.6–15.0)	0.580
Platelets G/L; median (range) Evaluable = 57	102 (5–1148)	93 (12–695)	107 (5–1148)	0.800
PB blasts %; median (range) Evaluable = 55	0 (0–57)	0 (0–57)	0 (0–18)	0.330

*PB* peripheral blood, *Hypergamma *hypergammaglobulinemia

**Table 5:** Molecular features stratified by the presence or absence of hypergammaglobulinemia

Mutated gene VAF (≥ 5%)	With hypergammaglobulinemia	Without hypergammaglobulinemia	*P*-value
*NRAS*	4/14 (29%)	1/23 (4%)	0.037
*KRAS*	2/14 (14%)	3/23 (13%)	0.915
*CBL*	3/14 (21%)	3/23 (13%)	0.502
*NF1*	0/11 (0%)	4/17 (24%)	0.082
*PTPN11*	1/14 (7%)	3/23 (13%)	0.575
*TET2*	8/14 (57%)	20/24 (83%)	0.077
*IDH1/2*	2/14 (14%)	0/24 (0%)	0.057
*ASXL1*	4/14 (29%)	10/23 (43%)	0.365
*EZH2*	0/14 (0%)	4/24 (17%)	0.106
*DNMT3A*	0/14 (0%)	3/24 (13%)	0.168
*SRSF2*	5/14 (36%)	8/24 (33%)	0.881
*ZRSR2*	0/14 (0%)	6/24 (25%)	0.041
*U2AF1*	1/14 (7%)	2/23 (13%)	0.867
*SF3B1*	0/14 (0%)	2/24 (8%)	0.267
*RUNX1*	0/14 (0%)	5/24 (21%)	0.067
*TP53*	5/14 (36%)	5/20 (25%)	0.500

*VAF* variant allele frequency

## Discussion

Serum electrophoresis is an excellent tool for analyzing the inflammation status in patients. In the early phase of acute inflammation, the α1 and α2 fractions are increased, whereas changes in albumin and the γ fraction are not regularly seen [25]. In the late phase of acute inflammation, the serum albumin starts to decrease and the γ fraction to increase. Chronic inflammatory disorders are characterized by increased gamma globulins and by decreased serum albumin, whereas the α1 and α2 fractions are not altered anymore.

Since serum electrophoresis is not routinely performed in myeloid disorders, it was available in only a subgroup of patients in our ABCMML database. In these patients, we have seen relative polyclonal hypergammaglobulinemia in 42%. There was a clear inverse correlation to albumin levels, indicating opposite changes of gamma globulins and albumin in chronic inflammation. In a subgroup of patients, α1 and/or α2 fractions were increased in the electrophoresis, suggesting a persistent acute-phase pattern in these patients.

The mechanism behind hypergammaglobulinemia in CMML patients remains to be determined. Polyclonal hypergammaglobulinemia has already been described in previous series of patients with CMML [26]. We have looked for possible correlations of hypergammaglobulinemia with phenotypic and molecular features. Regarding laboratory features including leukocyte count, hemoglobin value, platelet value, and circulating blasts, we could not find any differences in patients with or without hypergammaglobulinemia, indicating that the mechanisms leading to changes in these parameters are different from the mechanism leading to hypergammaglobulinemia. Regarding molecular parameters, we observed an association of hypergammaglobulinemia and molecular aberrations of the *NRAS* and *ZRSFR* genes. These findings are unexpected, but have to be considered with caution due to the small number of CMML patients who had molecular analysis. However, in this context, it has to be mentioned that recently, a functional link between molecular aberrations and activation of the inflammasome was reported in a preclinical model [27]. In this mouse model, Kras-driven myeloproliferation was reversed by functional inactivation of NLRP1, a major component of the inflammasome. A similar phenotypic improvement was seen with therapeutic IL‑1 receptor blockade.

Most strikingly, patients with hypergammaglobulinemia had inferior survival as compared to CMML patients without hypergammaglobulinemia. This adverse impact on survival was found in univariate analysis and was retained in the multivariate analysis in the presence of thrombocytopenia, which was the only adverse blood parameter, indicating that the pathophysiologic basis for the two alterations are different. In larger series, all parameters including leukocytosis, anemia, thrombocytopenia, and circulating blast cells are established prognostic parameters. The lack of prognostic impact of these parameters, except thrombocytopenia, is most likely the result of the small patient cohort in this study. Therefore, the prognostic impact of hypergammaglobulinemia in this study deserves even more attention. There is clear evidence in the literature that IL‑6 is one of the main stimulatory factors of IgG production [28, 29]. On the other hand, it has been shown that CMML cells can produce IL‑6 in vitro and stimulate CMML cell growth in an autocrine manner [30]. One attractive hypothesis of our exploratory findings could be that IL‑6 may provide a common basis for hypergammaglobulinemia and the adverse outcome in these patients. This hypothesis must be further explored in future studies.

We are aware of the limitations of our study. For example, most of the information used in this study was derived from retrospective real-world data that were not collected systematically or prospectively. Thus, not every parameter was available in all patients. In addition, data from patient records were obtained over many years and from many different centers. Moreover, the patients included in this study were a relatively heterogenous population regarding the blast cell counts, and there was a lack of molecular data in a significant number of patients. However, real-world data have recently been recognized as an important way to get insights into the routine management and natural history of rare diseases [31]. CMML is a rare disease and adequate patient numbers for a systematic and prospective study are not easy to collect within a limited timeframe. Moreover, the ABCMML provides information derived from molecular as well as from functional studies and therefore allows a more comprehensive view and deeper insight into the complex pathophysiology of this hematologic malignancy [22].
